# Local lung coagulation post resection: an ex-vivo porcine model

**DOI:** 10.1007/s10103-021-03280-7

**Published:** 2021-03-23

**Authors:** A. Kirschbaum, Th. M. Surowiec, A. Pehl, Th. Wiesmann, D. K. Bartsch, N. Mirow

**Affiliations:** 1 Department of Visceral, Thoracic and Vascular Surgery, University Hospital Marburg, Marburg, Germany; 2 Department of Mathematics and Computer Science, University of Marburg, Marburg, Germany; 3 Institute of Pathology, University Hospital Marburg, Marburg, Germany; 4 Department of Anesthesiology and Intensive Care Medicine, University Hospital Marburg, Marburg, Germany; 5 Department of Cardiac and Thoracic Vascular Surgery, University Hospital Marburg, Marburg, Germany

**Keywords:** Coagulation, Nd:YAG laser, Non-anatomical resection, Laser bare fiber, Pulmonary air leakage

## Abstract

Following non-anatomical resection of lung parenchyma with a Nd:YAG laser, a coagulated surface remains. As ventilation starts, air leakage may occur in this area. The aim of the present study was to investigate, whether additional coagulation either before or after ventilation has an additional sealing effect. Freshly slaughtered porcine heart-lung blocks were prepared. The trachea was connected to a ventilator. Using a Nd:YAG laser (wavelength: 1320 nm, power: 60 W), round lesions (1.5 cm in diameter) with a depth of 1.5 cm were applied to the lung using an 800-μm laser fiber (5 s per lesion). Group 1 (*n* = 12) was control. Additional coagulation was performed in group 2 (*n* = 12) without and in group 3 (*n* = 12) with ventilation restarted. Air leakage (ml) from the lesions was measured. The thickness of each coagulation layer was determined on histological slices. Differences between individual groups were analyzed by one-way ANOVA (significance *p* < 0.05). After resection, 26.2 ± 2.7 ml of air emerged from the lesions per single respiration in group 1. Air loss in group 2 was 24.6 ± 2.5 ml (*p* = 0.07) and in group 3 23.7 ± 1.8 ml (*p* = 0.0098). In comparison to groups 1 and 2 thickness of the coagulation layers in group 3 was significantly increased. After non-anatomical porcine lung resection with a Nd:YAG laser, additional coagulation of the ventilated resection area can reduce air leakage.

## Introduction

Lung metastases are often removed non-anatomically using a Nd:YAG laser [1, 2], whereas pulmonary metastases are often removed via open access (thoracotomy). During open surgery the entire lung is carefully palpated. Following the technical trend minimally invasive procedures are increasingly favored, if technically feasible. In these procedures for non-anatomical resection of lung metastases thin laser fibers can be employed under thoracoscopic view [3]. After resection a coagulated resection surface remains bearing the risk of bleeding and air leakage. Therefore, in clinical routine frequently the resection area is additionally coagulated by laser. However, evidence regarding procedural risk or benefit of this procedure is scarce. In the center of a cutting lesion surgical lasers generate intense heat leading to tissue vaporization. However, near the margins of the vaporization zone the temperature is significantly lower and the lung tissue is merely coagulated. Depending on the energy of the laser, thickness of this coagulation zone may differ.

If coagulation of blood vessels is complete, a bloodless operating site may ensue [4, 5]. Especially in superficial resections [6] coagulation layers may well seal the lung parenchyma from air leaks. However, with increasing resection depth there is a growing risk of opening small bronchi or even segmental bronchi. The latter openings are clearly visible upon inspection of the resection surfaces and will lead to considerable air loss [6]. To avoid the risk of air leakage after laser resection in surgery, the majority of surgeons will routinely close resection sites by simple over-sutures. For deeper resections, several layers of sutures are used to avoid intrapulmonary cavitation and the potential risk of infection. On the other hand as a consequence of multiple laser resections and suturing, considerable, even functionally relevant restriction of the lung may develop [5]. Avoiding parenchymal suturing wherever possible therefore improves operative results.

Further sealing of resection surfaces by repeated laser coagulation is a possible option not yet sufficiently studied. The authors’ idea was that this will increase the thickness of an initial coagulation layer thereby improving airtightness.

In our study, we aimed at an experimental design to investigate the influence of lung ventilation during laser coagulation of pulmonary resection sites. In order to achieve good reproducibility, we opted for an ex-vivo model of porcine lungs.

## Materials and methods

From freshly slaughtered pigs (weight 90 kg EU standard) the heart and both lungs were taken en bloc. As specimen were exclusively obtained from animals slaughtered for nutritional purposes, ACUC approval for animal studies or related body was not required.

The trachea was severed below the larynx. Remnants of the diaphragm and pericardium were removed. Preparations were examined for their external integrity and those with lesions on the lungs were discarded. Immediate transport to our laboratory followed. Only intact preparations were used for the experiments.

**Fig. 1:**
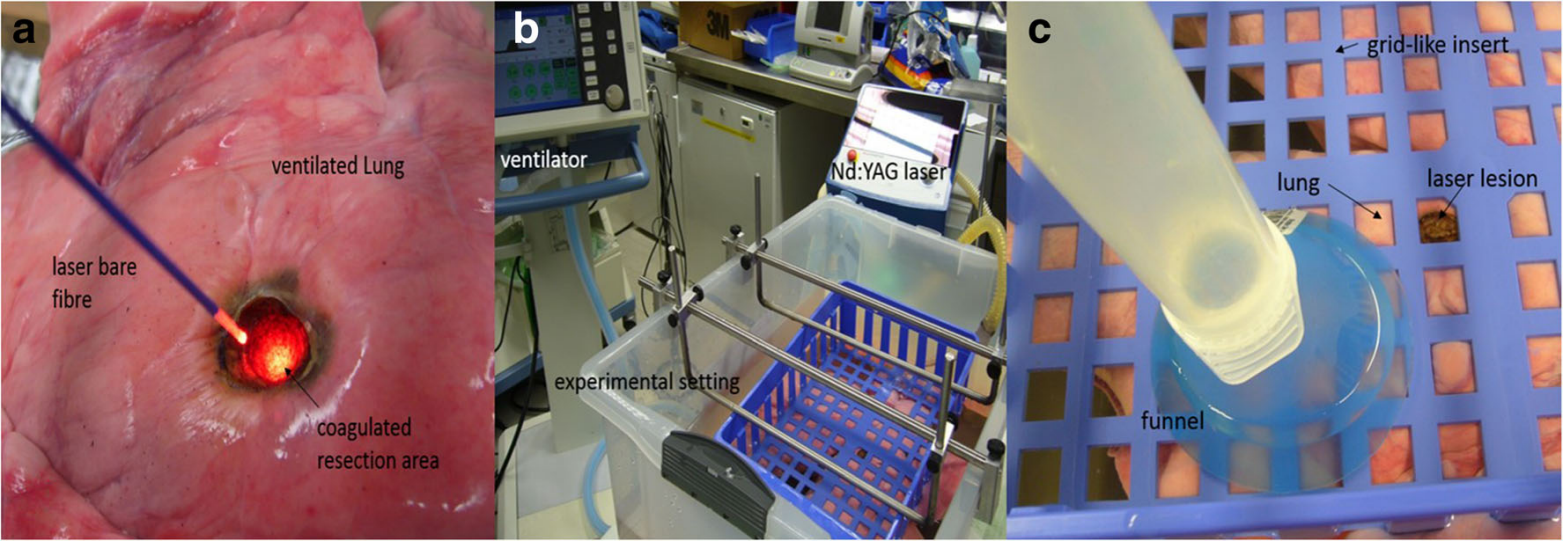
**a** Additional coagulation of the resection surface by laser fiber (power 60 W) in the ventilated lung. **b** Overview of the experimental setup. The ventilated lung is fixed by a grid. **c** To determine air leakage in ml/respiration, a funnel is pushed over the laser lesion. The ascending air (ml) is indicated on the volumetric flask

The trachea was intubated with a tube (Vygon 520, CH 8.0, Braun, Melsungen, Germany), and the tube balloon was blocked in the trachea. The tube was connected to a ventilator (Cicero EM PM 8060, Dräger, Lübeck, Germany), fixed to a board, and the entire preparation placed into a waterproof container. The intended lesions on the lung surface were marked by a stamp (diameter: 1.5 cm) and ventilation was stopped. All cylindrically shaped resections with a depth of 1.5 cm were performed with a Nd:YAG Laser LIMAX® 120 (wavelength: 1320 nm) (Gebrüder KLS Martin & Co., KG, Tuttlingen, Germany) and an 800-μm laser fiber at a laser power of 60 W (see Fig. 1a).

After resection, the lungs were ventilated. A peak inspiratory pressure of 25 mbar with a PEEP of + 5 mbar was chosen, this being equivalent to clinically employed pressures for determination of intraoperative air loss [7, 8]. Ventilation frequency was set to 10/min. To quantify air loss across the lesions, the lungs were submerged under water and held by a grid-like insert (see Fig. 1b). Lesions created by the laser were positioned underneath the grid openings. Air escaping from the lesions per single respirations was collected via a funnel filled with water attached to a volumetric flask (see Fig. 1c). Measurements were repeated five times for all lesions created and means were calculated.

We initially performed 12 resections without further intervention (group 1). In a second experiment additional coagulation of the lesions was performed with a laser power of 60 W at a distance of 1 cm to the lung surface for 5 s. Depending on the protocol, coagulation was carried out either before (group 2) or after renewed ventilation (group 3).

Tissue samples were taken from the resection areas for histological evaluation and sections were subjected to hematoxylin-eosin staining (HE). Thickness of coagulation zones (in μm) was measured using the program Image J version 1.46r (National Institutes of Health, Bethesda, USA).

Air losses of the individual groups were calculated via a one-way ANOVA and pairwise multiple comparison procedures (Holm–Sidak method). Statistical significance was set at *p* < 0.05. Regarding histological thickness of the coagulation layers groups were compared by non-parametric Mann Whitney *U* test (significance was set at *p* < 0.05).

The software package Graph Prism version 6.0 (GraphPad Software Inc., La Jolla, CA, USA) was used for statistical analysis and creation of the graphs.

## Results

**Fig. 2:**
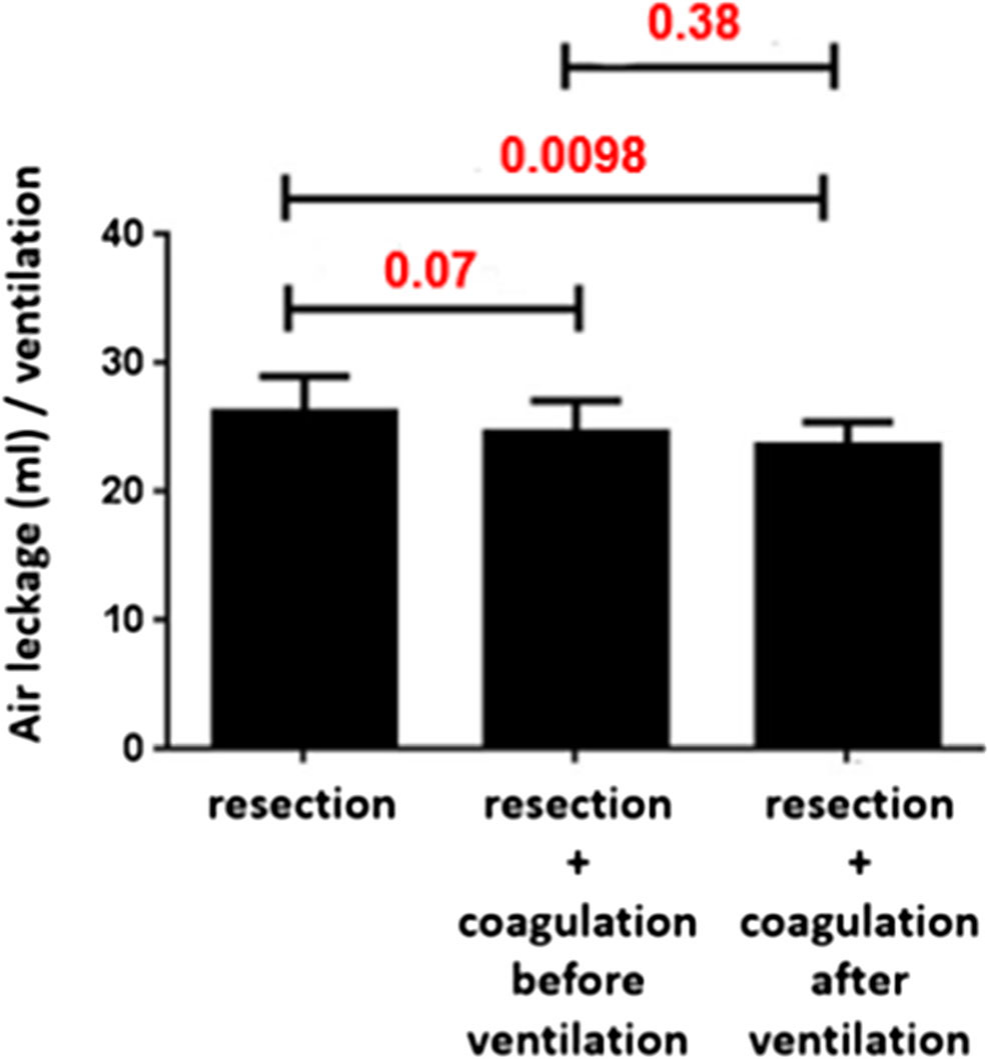
Comparison of individual groups with regard to mean air leakage per respiration

In group 1 (no additional coagulation) an average air loss of 26.2 ± 2.7 ml per single respiration was determined. For this group the average thickness of the coagulation layer was determined to be 111.9 ± 1.5 μm. For group 2 (additional coagulation without ventilation) we calculated an air loss of 24.6 ± 2.5 ml after ventilation. Histological evaluation of the coagulation layer revealed an average thickness of 113.1 ± 1.4 μm. In group 3 (coagulation during renewed ventilation) mean air loss was 23.7 ± 1.8 ml. The histologically determined mean thickness of coagulation layers was 145.0 ± 2.2 μm (see Table 1 and Fig. 2). In all three groups the pattern of air leakage was observed in the form of continuous chains of bubbles.

**Table 1:** Comparison of air leakage and thickness of coagulation layer between groups

	Group 1: resection without coagulation	Group 2: resection + coagulation without ventilation	Group 3: resection + coagulation with ventilation
*n*	12	12	12
ml per respiration Mean ± SD	26.2 ± 2.7	24.6 ± 2.5	23.7 ± 1.8
Thickness of the coagulation layer (μm) Mean ± SD	111.9 ± 1.5	113.1 ± 1.4	145.0 ± 2.2

**Fig. 3:**
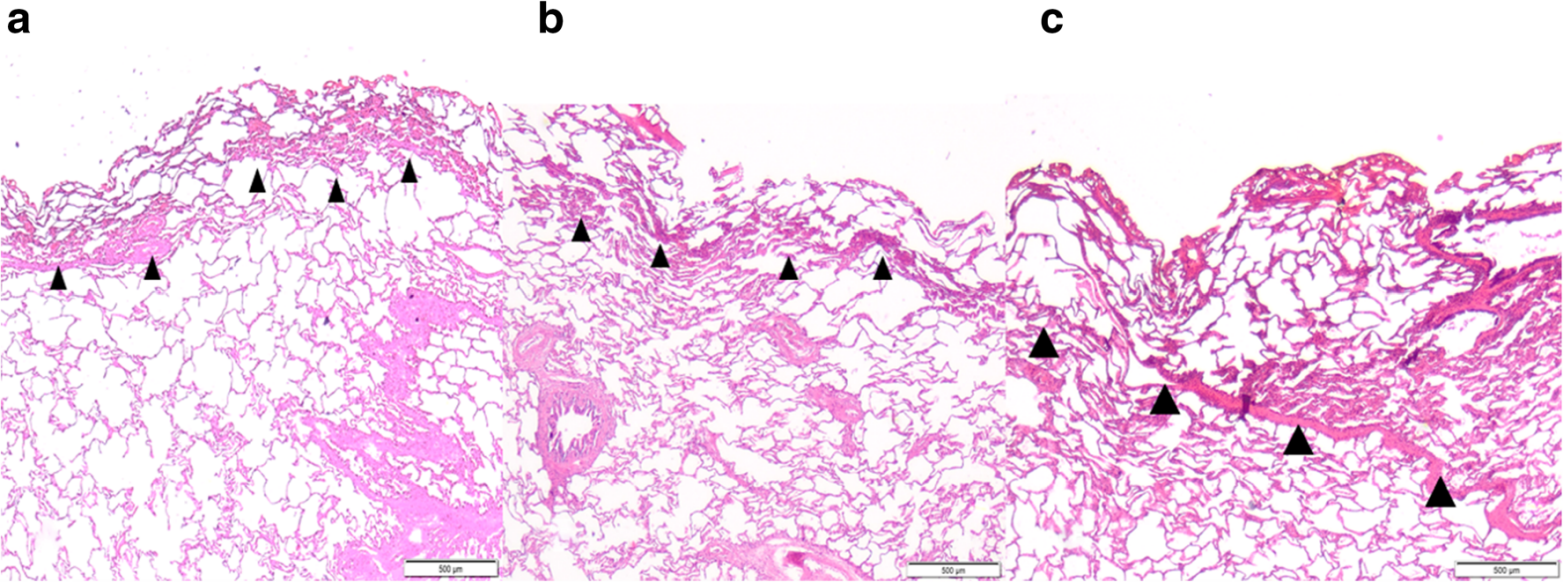
Histological sections of the resection surfaces (HE staining). Arrows mark the margins of the coagulation zone. **a** Resection with laser power of 60 W. **b** Resection with laser fiber 60 W, then coagulation for 5 s at 60 W respire without ventilation. **c** Resection with laser fiber 60 W, then coagulation at 60 W for 5 s, ventilated lung. Magnification: 12.5-fold

Comparing the quantity of air lost, groups 1 and 2 did not differ significantly (*p* = 0.07). Similarly, there was no significant difference between groups 2 and 3 (*p* = 0.38) (see Tables 2 and 3). However, a significant difference was found between groups 1 and 3 (*p* = 0.0098). Looking at mean thicknesses of the coagulation layer, groups 1 and 2 were not significantly different (*p* = 0.06). In contrast, a highly significant difference (*p* < 0.0001) was found between groups 1 and 3 as well as between groups 2 and 3 (see Fig. 3).

**Table 2:** Air leakage: one-way analysis of variance^a^

**Group**	***n***	**Missing**	**Mean**	**SD**	**SEM**
1	12	0	25.25	2.563	0.74
2	12	0	24.5	2.541	0.733
3	12	0	23.67	1.723	0.497
**Source of variance**	**DF**	**SS**	**MS**	***F***	***p***
Between groups	2	41.72	20.861	3.913	0.03
Residual	33	175.917	5.331		
Total	35	217.639			

^a^Normality test passed (*p* = 0.067) and equal variance test passed (*p* = 0.501). The differences in the mean values among the treatment groups are greater than would be expected by chance; there is a statistically significant difference (*p* = 0.03)

*DF* degrees of freedom, *MS* means of squares, *SEM* standard error of the mean, *SS* sums of square resection + coagulation before ventilation

**Table 3:** Air leakage: pairwise multiple comparison of all procedures^a^

Comparison	Difference of means	*T*	Unadjusted *p*	Critical level
Group 1 vs. 3	2.583	2.741	0.00982	0.017
Group 1 vs. 2	1.75	1.857	0.0723	0.025
Group 2 vs. 3	0.833	0.884	0.382	0.05

^a^Calculated by Holm-Sidak method; overall significance level *p* = 0.05

## Discussion

Airtightness after non-anatomical parenchymal lung resection with an Nd:YAG laser is a highly relevant clinical issue. Air leakage not only causes a prolonged chest drainage period but may also lead to longer inpatient treatment. Furthermore, in case of persistent air loss, operative revision of the leaking lung may be needed [9, 10]. Prolonged periods of chest tubes in situ in addition are associated with an increased risk of infection [9, 11]. Consequently, many thoracic surgeons always seal resection surfaces with sutures [12]. However, as additional suturing bears the risks of restriction and infection, effective coagulation may perhaps be the better alternative.

In a previous study [6] we were able to show that in superficial laser resections coagulation zones are sufficiently airtight. In deeper resections thickness of the coagulation layers are laser energy dependent [4].

If resection surfaces were coagulated in the ventilated lung, a significant decrease in the amount of air leaking was observed. The effect was clearly visible upon inspection and objectively confirmed by quantitative measurement. Resulting coagulation layers were increased in thickness. In theoretical thought ventilation of the resection surfaces will stretch the tissue so a potential for rupture of the primary coagulation layer may ensue. The previously resected and therefore partly coagulated parenchyma including potentially ruptured sites are additionally coagulated so that a more stable and thicker coagulation layer is produced. Branscheid [13] in 1992 reported a positive effect of coagulation of the resection surface after laser application on the half-distended lung. Additional laser coagulation after laser resection is a rather uncomplicated procedure, which, in clinical practice, is easily performed via an open or thoracoscopic access. Judging from our experimental findings, additional coagulation may be useful up to a resection depth of 1.5 cm. In deeper lesions, opening of small segmental bronchi is likely. According to our previous study [6], these bronchi are transected in the process of parenchymal resection and their lumina cannot be closed by coagulation. Suturing of the resection surfaces is required.

There are several limitations to this study. Heat generation in tissue by a Nd-YAG laser is physically based on exciting chromophores. Among chromophores involved are water, hemoglobin, and oxy-hemoglobin. In our experiments lungs were not perfused, thus reducing especially hemoglobin and oxy-hemoglobin, possibly influencing results. Furthermore, we only examined initial airtightness and we have no information regarding persistence of the effects stated over time. Therefore, as our investigations were performed ex vivo and neither on living tissue nor on human organs, we are not drawing clinical consequences.

Nevertheless, our experimental data contribute to the existing knowledge and we anticipate that they will facilitate current modeling and simulation efforts within the research group for laser-tissue interactions. As a possible next step an in vivo model may be suitable. Modifications of coagulation, e.g., duration of application and variations in the ventilation state of the lungs, should be further investigated

## Conclusion

In an ex vivo porcine lung model, air loss via the resection surface after non-anatomical laser resection can be reduced by additional coagulation. Our data indicate that this should preferably be performed on the ventilated rather than on the non-ventilated lung.
